# CO_x_ hydrogenation to methanol and other hydrocarbons under mild conditions with Mo_3_S_4_@ZSM-5

**DOI:** 10.1038/s41467-023-36259-9

**Published:** 2023-01-31

**Authors:** Gui Liu, Pengfei Liu, Deming Meng, Taotao Zhao, Xiaofeng Qian, Qiang He, Xuefeng Guo, Jizhen Qi, Luming Peng, Nianhua Xue, Yan Zhu, Jingyuan Ma, Qiang Wang, Xi Liu, Liwei Chen, Weiping Ding

**Affiliations:** 1 Key Lab of Mesoscopic Chemistry, School of Chemistry and Chemical Engineering, Nanjing University, Nanjing, China; 2 Department of Applied Chemistry, School of Chemistry and Molecular Engineering, Nanjing Tech University, Nanjing, China; 3 i-Lab, CAS Centre for Excellence in Nanoscience, Suzhou Institute of Nano-Tech and Nano-Bionics, Chinese Academy of Sciences, Suzhou, PR China; 4 Shanghai Synchrotron Radiation Facility, Pudong New District, Shanghai, China; 5 School of Chemistry and Chemical, In-situ Centre for Physical Sciences, Frontiers Science Centre for Transformative Molecules, Shanghai Jiao Tong University, Shanghai, PR China

**Keywords:** Heterogeneous catalysis, Porous materials, Energy

## Abstract

The hydrogenation of CO_2_ or CO to single organic product has received widespread attentions. Here we show a highly efficient and selective catalyst, Mo_3_S_4_@ions-ZSM-5, with molybdenum sulfide clusters ([Mo_3_S_4_]^n+^) confined in zeolitic cages of ZSM-5 molecular sieve for the reactions. Using continuous fixed bed reactor, for CO_2_ hydrogenation to methanol, the catalyst Mo_3_S_4_@NaZSM-5 shows methanol selectivity larger than 98% at 10.2% of carbon dioxide conversion at 180 °C and maintains the catalytic performance without any degeneration during continuous reaction of 1000 h. For CO hydrogenation, the catalyst Mo_3_S_4_@HZSM-5 exhibits a selectivity to C_2_ and C_3_ hydrocarbons stably larger than 98% in organics at 260 °C. The structure of the catalysts and the mechanism of CO_x_ hydrogenation over the catalysts are fully characterized experimentally and theorectically. Based on the results, we envision that the Mo_3_S_4_@ions-ZSM-5 catalysts display the importance of active clusters surrounded by permeable materials as mesocatalysts for discovery of new reactions.

## Abstract (web summary)

A series of materials containing Mo-S clusters confined in zeolitic cages of ZSM-5 are reported and shown to be efficient for CO_2_ or CO hydrogenation with >98% selectivity to methanol, stable over 1000 h, or C_2_ and C_3_ hydrocarbons, stable over 100 h.

## Introduction

Methanol is one of the most important commodity chemicals and usually prepared in industry by syngas conversion over Cu/ZnO/Al_2_O_3_ catalysts at elevated temperatures (230-300 °C) and pressures (5-10 MPa)^1, 2^. As an alternative, the hydrogenation of CO_2_ is also an effective route to methanol synthesis and, as shown in Eqs. (1)-(3), the utilization of chemical energy contained in feed gases of the two routes is in fact similar. Although the hydrogenation of CO_2_ to methanol, at present time, seems to face some challenges in catalyst activity, selectivity and stability, the intensive study on the reaction would provide a more efficient route for industrial production of methanol and utilization of CO_2_^3, 4^. For the process, however, the side reaction of reverse water-gas shift (RWGS, Eq. 3), is almost unavoidable and, the higher the temperature, the more serious the RWGS reaction, due to its endothermic nature. It not only lowers selectivity toward methanol, but also intensifies the sintering of the active phase at higher temperature and reduce the stability of the catalyst, due to the by-product water^5^. How to avoid the occurrence of RWGS reaction has been a troublesome question for a long time.

1 $${{{\mbox{CO}}}}_{2}+3{{{\mbox{H}}}}_{2}\to {{\mbox{C}}}{{{\mbox{H}}}}_{3}{{\mbox{OH}}}({{\mbox{g}}})+{{{\mbox{H}}}}_{2}{{\mbox{O}}}({{\mbox{g}}})\bigtriangleup \!{{\mbox{H}}}=-49{{\mbox{.}}}5{{\mbox{kJ/mol}}}$$

2 $${{\mbox{CO}}}+2{{{\mbox{H}}}}_{2}\to {{\mbox{C}}}{{{\mbox{H}}}}_{3}{{\mbox{OH}}}({{\mbox{g}}})\bigtriangleup \!{{\mbox{H}}}=-90{{\mbox{.}}}1{{\mbox{kJ/mol}}}$$

3 $${{\mbox{C}}}{{{\mbox{O}}}}_{2}+{{{\mbox{H}}}}_{2}\to {{\mbox{CO}}}+{{{\mbox{H}}}}_{2}{{\mbox{O}}}\bigtriangleup \!{{\mbox{H=}}}41{{\mbox{.}}}1{{\mbox{kJ/mol}}}$$

4 $${{\mbox{C}}}{{{\mbox{O}}}}_{2}+4{{{\mbox{H}}}}_{2}\to {{\mbox{C}}}{{{\mbox{H}}}}_{4}+2{{{\mbox{H}}}}_{2}{{\mbox{O}}}\bigtriangleup \!{{\mbox{H}}}=-164{{\mbox{.}}}9{{\mbox{kJ/mol}}}$$

The thermodynamic calculation on reactions 1, 3, and 4 with mixture of CO_2_/3H_2_ as feed gases indicates the RWGS reaction is negligible at temperatures lower than 180 °C, as shown in Supplementary Fig. 1, and the formation of CH_4_ has large advantages in thermodynamics, especially at low temperatures. For products of oxygenates from CO_2_ hydrogenation, the catalyst must be active for cleavage of the first C-O bond but inactive for the second C-O bond of CO_2_. It is a great challenge to develop such a catalyst effective for CO_2_ hydrogenation at temperatures lower than 180 °C.

In other aspects, the hydrogenations of CO_2_ and CO have complicated multi-relation. The product distribution of CO hydrogenation (Fischer-Tropsch synthesis, FTS) generally follows the famous Anderson-Schulz-Flory (ASF) law. Breaking through the limitations of ASF model for FTS reactions to obtain single or several products has been of particular importance and long term desired^6–8^. To synthesize methanol via CO_2_ hydrogenation reaction in near exclusive selectivity is very meaningful and the mechanism elucidated can offer basises to develop efficient catalysts for synthesis of low hydrocarbons in specially high selectivity.

In recent years, the catalysts active for CO_2_ hydrogenation have been investigated widely and the most valued catalysts would be Cu/ZnO/Al_2_O_3_^9–12^ and indium oxide-based catalysts^3, 5, 13^. For Cu/ZnO/Al_2_O_3_ catalyst, the main problem would be the unavoidable RWGS reaction which lowers methanol selectivity. Other copper-based catalysts such as Cu/ZnO^14^, Cu/CeO_x_/TiO_2_^1^ and Cu/ZrO_2_^15^ often give the selectivity to methanol less than 70% in hydrogenation of CO_2_. Indium oxide-based catalysts have been studied intensively and have shown great potential in the hydrogenation of CO_2_ to methanol. A 7% conversion of CO_2_ and 40% selectivity toward methanol can be achieved with pure In_2_O_3_ catalyst under the reaction conditions of 330 °C and 5 MPa^16^. A selectivity to methanol > 99% over In_2_O_3_/ZrO_2_ catalyst has been reported with CO_2_ conversion of only ~5%^4^. In addition to copper and indium oxide-based catalysts, ZnO-ZrO_2_ composite oxide catalyst has also made great progress in the hydrogenation of CO_2_ to methanol, which achieved 86% selectivity toward methanol and more than 10% conversion of CO_2_ at 5 MPa and 320 °C^17^.

We have been impressed for a long time by unique catalytic properties of the composite composed of ZSM-5 zeolite and its intracrystalline tough clusters of metal compounds, such as MoN_x_@ZSM-5^18^, Pt_x_@ZSM-5^19^, and Iglesia’s work on MoC_x_@ZSM-5^20^ and WC_x_@ZSM-5^21^. The strong regulatory effect of ZSM-5 on the confined clusters and the effect of cooperations among the clusters, the zeoliti acidic sites and the zeolitic porosity on conversions of reactive molecules lead to the catalytic property of the composite unpredictable and extremely interesting. In this work, we report a highly efficient catalyst combined [Mo_3_S_4_]^n+^ clusters as active centers and porous NaZSM-5 as confining or peripheral framework for the hydrogenation of CO_2_ to methanol in fixed bed reactor (Mo: 3 wt%, Mo_3_S_4_: 4.3 wt%). Interestingly, our current study is related to recent report by Wang and Deng^22^, where they have reported a catalyst of few-layer MoS_2_ with sulfur vacancy for the hydrogenation of CO_2_ to methanol, showing similar catalytic performance but in different mechanism. The S/Mo ratio of current Mo_3_S_4_@NaZSM-5 catalyst is much lower than that of MoS_2_. More interestingly, when the Mo_3_S_4_@HZSM-5 (with proton as movable equilibrium ions) catalyst is used for syngas (2CO/H_2_) conversion, noteworthy that it exhibits a very stable selectivity to C_2_ and C_3_ hydrocarbons > 98% at 260 °C, or ~ 90% to C_2_-C_4_ hydrocarbons at 400°C with conversion of CO ~ 20%, indicative of the rich catalytic properties of the catalysts Mo_3_S_4_@ions-ZSM-5 for CO_x_ conversion.

## Results

### Structure and physicochemical properties of the catalysts

**Fig. 1:**
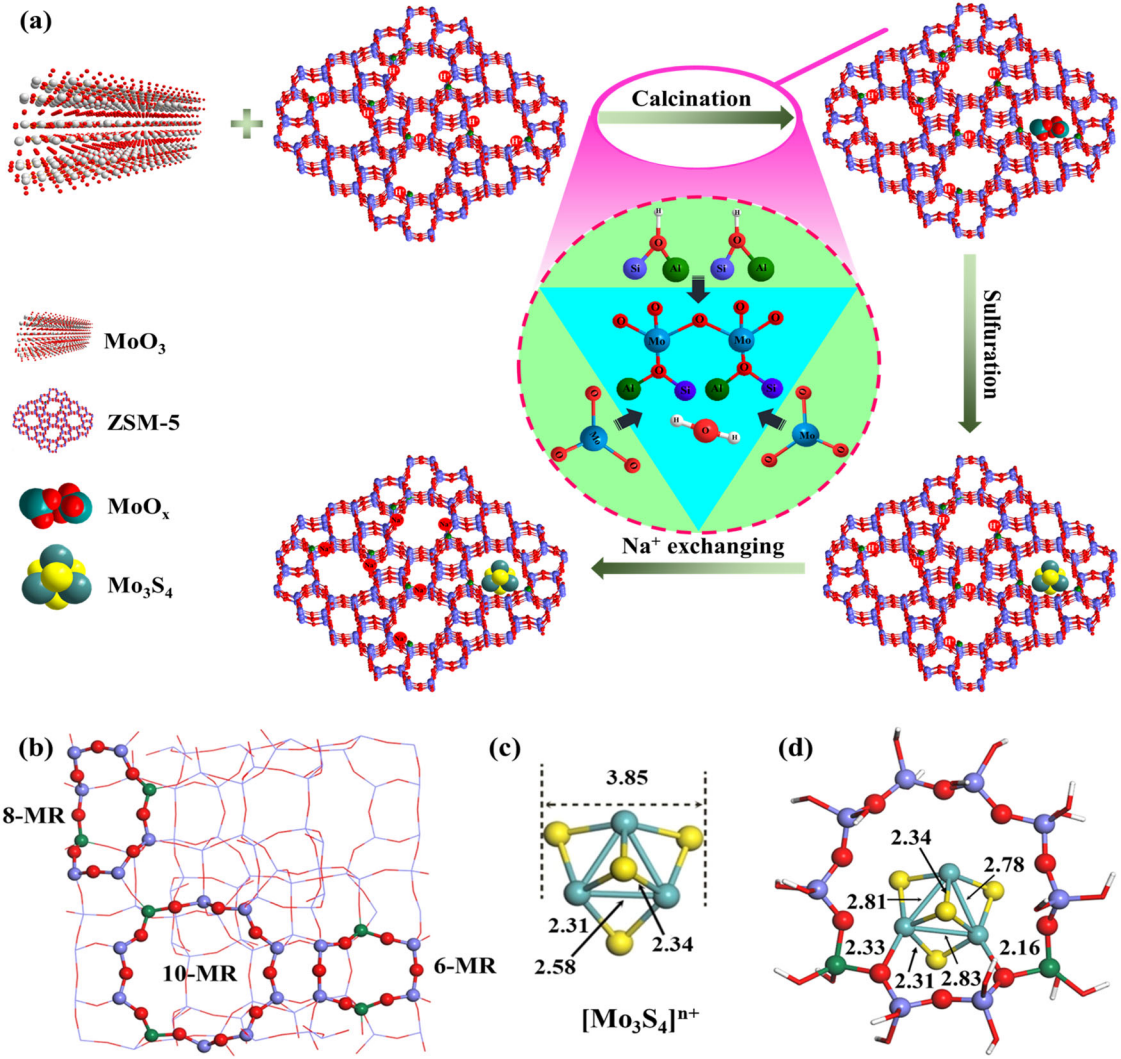
Schematic diagram of catalyst synthesis and structure optimized by DFT calculations. **a** Preparation of Mo_3_S_4_@NaZSM-5 (the shaded region refers to the scheme of solid exchange of MoO_3_ with OH groups in HZSM-5, the positions of aluminum and proton are schematically shown.); **b** The zigzag 10-MR(T3/T3), 8-MR(T7/T12), and δ-type 6-MR(T11/T11) Al-pair sites in the ZSM-5 framework; **c** Optimized structure of [Mo_3_S_4_]^n+^; **d** The optimized configuration of [Mo_3_S_4_]^n+^ intercalated into ZSM-5 molecular sieves containing double aluminum sites in 10-MR. Color legend: Si (purple), Al (green), O (red), Mo (Cyan), and S (yellow).

The Mo_3_S_4_@NaZSM-5 catalyst with [Mo_3_S_4_]^n+^ positioned in cages of ZSM-5 was synthesized by two-step mechanical mixing and calcination method, followed by neutralization of the zoelitic acidic sites in 0.05 M aqueous solution of NaOH, as schematically shown in Fig. 1a (details see Methods section). The structure of a ZSM-5 cage (Fig. 1b) containing a [Mo_3_S_4_]^n+^ cluster (Fig. 1c) was calculated and optimized using DFT method and the result was depicted in Fig. 1d, which is highly reliable to be described as Mo_3_S_4_@NaZSM-5, in accordance with the results of X-ray absorption fine structure (XAFS) and X-ray photoelectron spectroscopy (XPS) characterizations (Fig. 3).

**Fig. 2:**
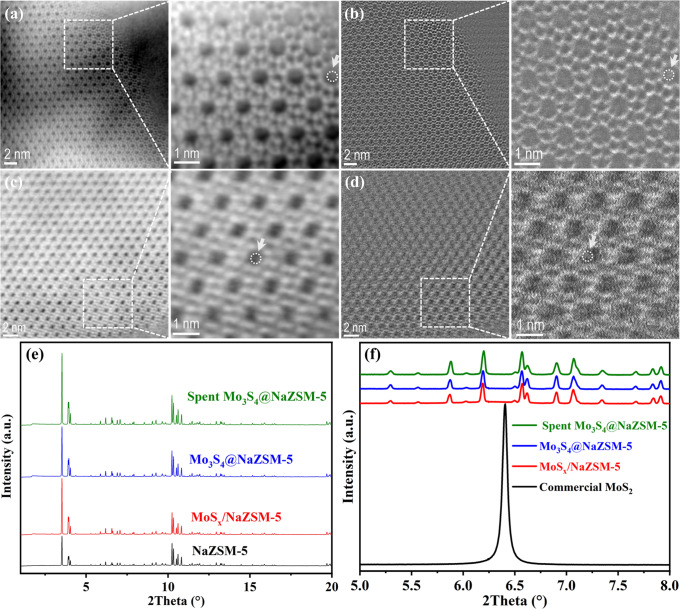
Structural characterizations of catalysts. **a** Scanning Transmission Electron Microscope-Integrated Differential Phase Contrast (STEM-iDPC) image of fresh Mo_3_S_4_@NaZSM-5 and corresponding enlarged view from the dotted area; **b** Scanning Transmission Electron Microscope-High Angle Annular Dark Field (STEM-HAADF) images of the identical area shown in Fig. 2a and corresponding enlarged view from the dotted area, both STEM-iDPC image and STEM-HAADF image were captured in the same area simultaneously; **c** STEM-iDPC image of the used Mo_3_S_4_@NaZSM-5 sample and corresponding enlarged view from the dotted area; **d** STEM-HAADF image of the used Mo_3_S_4_@NaZSM-5 sample and corresponding enlarged view from the dotted area; Filled clusters inside the 10 membered ring channels of ZSM-5 zeolite are surrounded by dotted white circles and pointed by white arrows, both STEM-iDPC image and STEM-HAADF image were captured in the same area simultaneously. **e** XRD patterns of NaZSM-5, MoS_x_/NaZSM-5, Mo_3_S_4_@NaZSM-5, and Spent Mo_3_S_4_@NaZSM-5; **f** Enlarged view of XRD patterns of commercial MoS_2_, MoS_x_/NaZSM-5, Mo_3_S_4_@NaZSM-5, and Spent Mo_3_S_4_@NaZSM-5 in the range of 5–8°.

The microstructures of the fresh and spent Mo_3_S_4_@NaZSM-5 catalysts were observed using Probe-Corrected Scanning Transmission Electron Microscope (STEM). As depicted in Fig. 2a–d, the typical framework of ZSM-5 of all samples remains unchanged, regardless with the incorporation of [Mo_3_S_4_]^n+^ into ZSM-5 zeolite or not, and the results of X-ray diffraction (XRD, Fig. 2e) also confirm the zeolite framework unchanged for all samples. The 10 membered ring of straight cylindrical channel opening to external of ZSM-5 zeolite can be clearly seen in the STEM images, with the pore diameters of about 0.55 nm. Combining Integrated Differential Phase Contrast (iDPC, to image both heavy and light atoms at low irradiation dose) and High-Angle Annular Dark Field (HAADF, with heavier atoms in higher brightness) imaging technology to directly image the zeolite and its pore filler^23^, it is confirmed that the ZSM-5 zeolite is indeed filled with molybdenum sulfide clusters, which are surrounded by dotted white circles and pointed by white arrows in enlarged view. The EDX profile obtained from the selected white square also confirms the presence of both S and Mo elements (Supplementary Fig. 2). It can be seen from STEM images that molybdenum sulfide clusters are in the size of around 0.4 nm and do not change or agglomerate before and after the reaction. For the MoS_x_/NaZSM-5 catalyst, however, it can be clearly seen from the HRTEM image that the lattice fringes correspond to layered MoS_2_ and the sizes of the MoS_2_ region are about ~10 nm, much larger than the channel of ZSM-5 zeolite, revealing the MoS_2_ are supported on the external surface of NaZSM-5 zeolite (Supplementary Fig. 3). As depicted in Fig. 2e, only the diffraction peaks from ZSM-5 zeolite can be seen in the XRD results of Mo_3_S_4_@NaZSM-5 and MoS_x_/NaZSM-5 catalysts. Even with the [Mo_3_S_4_]^n+^ clusters embedded into zeolitic pores, the crystallinity of Mo_3_S_4_@NaZSM-5 catalyst does not decay. The typical diffraction patterns related to MoS_x_ crystals could not be found in all samples (Fig. 2f), implying the highly dispersed state of MoS_x_. In addition, the diffraction patterns of the Mo_3_S_4_@NaZSM-5 catalyst after 1000 h on stream of CO_2_ hydrogenation are exactly the same as that of the fresh catalyst, indicative of the Mo_3_S_4_@NaZSM-5 catalyst is extremely stable under reaction conditions.

**Fig. 3:**
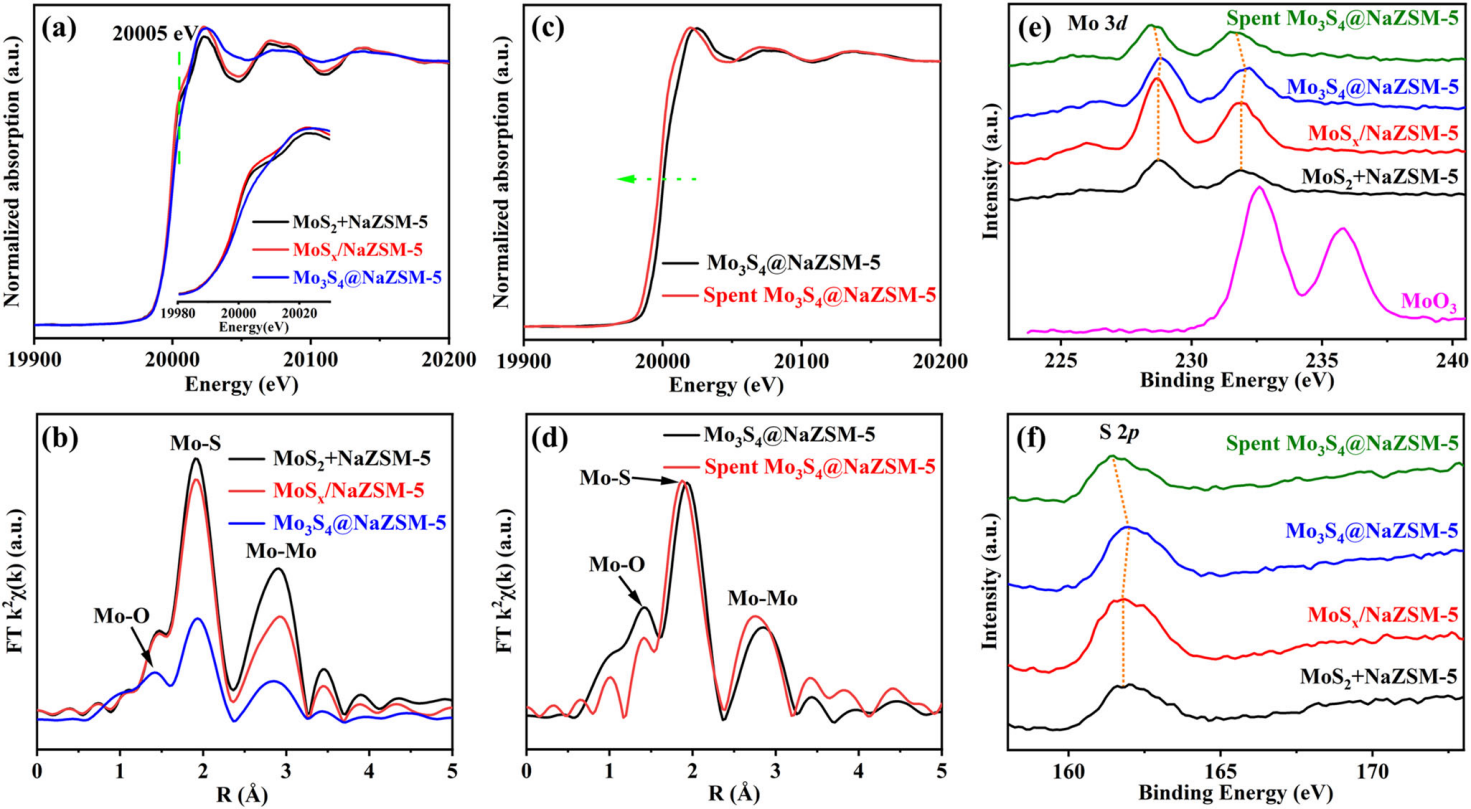
Structure and physical property of catalysts. **a** X-ray absorption near edge structure (XANES) spectroscopy and **b** radical distribution functions obtained by Fourier-transformed extended X-ray absorption fine structure (EXAFS) spectra of commercial MoS_2_, MoS_x_/NaZSM-5, and Mo_3_S_4_@NaZSM-5; **c** XANES spectroscopy and **d** radical distribution functions obtained by Fourier-transformed EXAFS spectra of Mo_3_S_4_@NaZSM-5 and spent sample; **e** Mo 3*d* XPS spectra of MoO_3_, commercial MoS_2_ + NaZSM-5, MoS_x_/NaZSM-5, Mo_3_S_4_@NaZSM-5, and Spent Mo_3_S_4_@NaZSM-5; **f** S 2*p* XPS spectra of commercial MoS_2_ + NaZSM-5, MoS_x_/NaZSM-5, Mo_3_S_4_@NaZSM-5, and Spent Mo_3_S_4_@NaZSM-5.

The coordination statuses of Mo or S in various samples were investigated using X-ray absorption fine structure (XAFS) spectroscopy. According to X-ray absorption near edge structure (XANES) measurements (Fig. 3a), the absorption of Mo K edge in Mo_3_S_4_@NaZSM-5 lacks a peak at a lower energy level (20005 eV), comparing with MoS_x_/NaZSM-5 and commercial MoS_2_ (the illustration for details), consistent with the literature results for [Mo_3_S_4_]^n+^ cluster^24^. The Mo K edge XANES of MoS_x_/NaZSM-5 is the same with that of commercial MoS_2_, implying that the MoS_x_ in MoS_x_/NaZSM-5 exists in the form of MoS_2_. The radial distribution functions of various samples obtained by Fourier-transformed extended X-ray absorption fine structure (EXAFS) spectra are shown in Fig. 3b. The coordination numbers and distances of Mo–S shell and Mo–Mo shell are obtained by fitting the radial distribution functions and listed in Table 1. Interestingly, the coordination numbers of Mo–S and Mo–Mo in Mo_3_S_4_@NaZSM-5 are much smaller than that of MoS_x_/NaZSM-5, but they are basically close to that of commercial MoS_2_ for MoS_x_/NaZSM-5. Combined with the HRTEM image of MoS_x_/NaZSM-5, shown in Supplementary Fig. 3, it can be concluded the external dispersion of MoS_2_ on NaZSM-5 zeolite for MoS_x_/NaZSM-5. The size of [Mo_3_S_4_]^n+^ clusters in Mo_3_S_4_@NaZSM-5, about 0.4 nm, is according to the coordination numbers and bond distances obtained through EXAFS measurement, which is also consistent with the results of STEM obervation. Moreover, the bonding of the Mo in [Mo_3_S_4_]^n+^ cluster to the framework O of NaZSM-5 zeolite (Fig. 3b)^25^ stabilizes the [Mo_3_S_4_]^n+^ cluster in the cage of NaZSM-5 zeolite, of which structure is theoretically optimized and shown in Fig. 1d. It is worthy of noting that the absorption of Mo K edge in spent Mo_3_S_4_@NaZSM-5 shifts slightly to a lower energy in comparison with fresh sample, due to the slight reduction or CH_x_ bonding to the Mo_3_S_4_@NaZSM-5 during hydrogenation reaction, as shown in Fig. 3c. These results are also in accordance with the Mo 3*d* binding energies measured using XPS for the spent Mo_3_S_4_@NaZSM-5, which move slightly to lower binding energies, compared with the fresh sample (Fig. 3e). Moreoer, it is interesting that both the Mo 3*d* and S 2*p* move slightly to lower binding energies, maybe due to intermediates of hydrocarbons adsorbed on the [Mo_3_S_4_]^n+^ cluster during hydrogenation reactions, as presented below. After 1000 h on stream at 180 °C, the coordination states of Mo and S and bond lengths in Mo_3_S_4_@NaZSM-5 have not changed and related EXAFS results are presented in Fig. 3d and Table 1, which fully shows that the Mo_3_S_4_@NaZSM-5 catalyst is extremely stable under typical reaction conditions, though the coordination of oxygen from zeolitic framework to the Mo clusters seems decreased a little after reaction, maybe caused by the tiny movement of the [Mo_3_S_4_]^n+^ clusters in channel of ZSM-5 which also results in the binding energy of Mo and S moving to lower positions.

**Table 1:** Curve-fitting results for various samples obtained by Fourier-transformed EXAFS spectra

Samples	Mo–S			Mo–Mo		
	N^a^	R (Å)^b^	σ^2^(Å^2^)^c^	N^a^	R (Å)^b^	σ^2^(Å^2^)^c^
Mo_3_S_4_@NaZSM-5	3.0 ± 0.2	2.332	0.0025	1.9 ± 0.4	2.931	0.0042
Spent Mo_3_S_4_@NaZSM-5	3.0 ± 0.3	2.310	0.0029	2.0 ± 0.3	2.93	0.0036
MoS_x_/NaZSM-5	5.7 ± 0.3	2.321	0.0027	4.8 ± 0.6	3.152	0.0045
MoS_2_ + NaZSM-5	6	2.321	0.0035	6	3.150	0.0039

^a^Coordination numbers.

^b^Bond distances.

^c^Debye-Waller factor.

The Mo 3*d* and S 2*p* XPS spectra of related samples are shown in Fig. 3e, f. For both the samples of Mo_3_S_4_@NaZSM-5 and MoS_x_/NaZSM-5 (Fig. 3e), the binding energies at about 232.6 and 235.8 eV of Mo 3*d*_5/2_ and Mo 3*d*_3/2_ related to MoO_3_ cannot be detected, suggesting the complete sulfurization of molybdenum after the Sulphur powder treatment. The Mo 3*d*_5/2_ and Mo 3*d*_3/2_ spectra of MoS_x_/NaZSM-5 are almost the same with that of the commercial MoS_2_ and the molybdenum is most likely in state close to Mo^4+^ species required by stoichiometry of MoS_2_^26^, in agreement with the assignment that the molybdenum exists in MoS_x_/NaZSM-5 just as MoS_2_ loaded on external surface and weakly interacted with the NaZSM-5. The catalyst Mo_3_S_4_@NaZSM-5 has much lower ratio of S/Mo, however, its Mo 3*d* binding energy values are higher than those of MoS_x_/NaZSM-5 and commercial MoS_2_, for the [Mo_3_S_4_]^n+^ clusters in Mo_3_S_4_@NaZSM-5 are stabilized in the pores of NaZSM-5 zeolite by the coordination of framework oxygen to Mo, as proved by Fourier-transformed EXAFS spectra of Mo_3_S_4_@NaZSM-5 (Fig. 3b). The bonding causes higher binding energy of Mo 3*d*_5/2_ and Mo 3*d*_3/2_ than that in MoS_x_/NaZSM-5 and commercial MoS_2_. The binding energy of Mo 3*d* in the spent Mo_3_S_4_@NaZSM-5 after 1000 h of reaction moves slightly to lower binding energy, compared with the fresh sample (Fig. 3e), indicating that the slight reduction or CH_x_ bonding to the Mo_3_S_4_@NaZSM-5 during hydrogenation reaction. As depicted in Fig. 3f, the XPS of S 2*p* exhibit trends basically consistent with that of Mo. Interestingly, although the molybdenum species in both catalysts Mo_3_S_4_@NaZSM-5 and MoS_x_/NaZSM-5 are completely sulfurized, the molar ratio of sulfur to molybdenum is very different. As shown in Supplementary Table 1, the molar ratio of sulfur to molybdenum in MoS_x_/NaZSM-5 is 1.89, which is close to that of the commercial MoS_2_ (1.90). However, the S/Mo ratio of Mo_3_S_4_@NaZSM-5 is 1.26, close to that of Mo_3_S_4_. These results indicate that the status of molybdenum and sulfur in MoS_x_/NaZSM-5 is similar to MoS_2_, while the molybdenum and sulfur in Mo_3_S_4_@NaZSM-5 are in totally different coordination forms. After 1000 h on stream of reaction, the mole ratio of S/Mo in the spent Mo_3_S_4_@NaZSM-5 is still close to 1.26 and no obvious desulfurization is found by XPS measurements.

The Mo contents in samples Mo_3_S_4_@NaZSM-5 and MoS_x_/NaZSM-5 were analyzed by inductively coupled plasma-optical emission spectrometer (ICP-OES) technique and the results are listed in Supplementary Table 2. The 3.01 and 3.03 wt% of Mo contents are obtained respectively for Mo_3_S_4_@NaZSM-5 and MoS_x_/NaZSM-5, close to the amount of molybdenum added in synthesis. Compared with NaZSM-5 and MoS_x_/NaZSM-5, Mo_3_S_4_@NaZSM-5 has the lowest Brunauer-Emmett-Teller (BET) specific surface area (Supplementary Table 2), indicating that [Mo_3_S_4_]^n+^ clusters occupy some zeolitic pores of ZSM-5 zeolite. While little difference is found between the specific surface area of MoS_x_/NaZSM-5 and NaZSM-5, consistent with the external loading of MoS_2_ on NaZSM-5 zeolite.

The confinement of metallic centers for hydrogenation by the zeolitic framework in catalyst Mo_3_S_4_@ZSM-5 is further checked by shape-selective hydrogenations of 2,3-dimethylnitrobenzene and nitrobenzene^19^^,^^27–31^ and the results are listed in Supplementary Fig. 4 and Supplementary Table 3, indicating that [Mo_3_S_4_]^n+^ is located in the interior of ZSM-5 zeolite for Mo_3_S_4_@ZSM-5, different from that of MoS_x_/ZSM-5.

### Catalytic hydrogenation of CO_2_

**Fig. 4:**
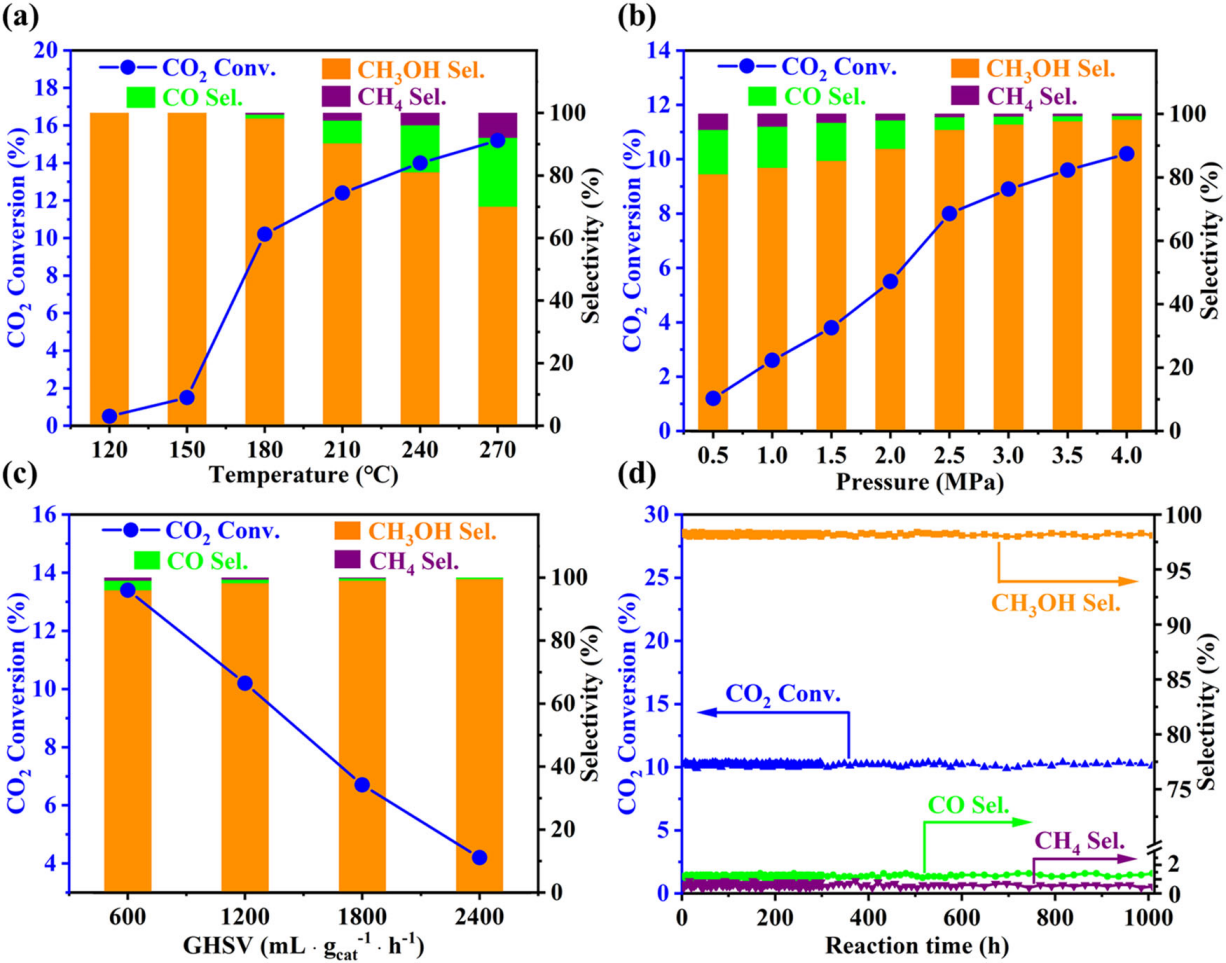
Hydrogenation of CO_2_ over Mo_3_S_4_@NaZSM-5 catalyst with feed gases of 23% CO_2_, 69% H_2_ and balance Ar. **a** Effect of reaction temperature (4 MPa, 1200 mL g_cat_^−1^ h^−1^); **b** Effect of reaction pressure (180 °C, 1200 mL g_cat_^−1^ h^−1^); **c** Effect of space velocity (180 °C, 4 MPa); **d** Stability test of the catalyst Mo_3_S_4_@NaZSM-5 (180 °C, 4 MPa, 1200 mL g_cat_^−1^ h^−1^). Conv. and Sel. are abbreviations for Conversion and Selectivity, respectively.

The catalytic properties of Mo_3_S_4_@NaZSM-5 catalyst for CO_2_ hydrogenation are measured and depicted in Fig. 4. As shown in Fig. 4a, the conversion of CO_2_ increases with the increase of reaction temperature from 120 °C to 270 °C. When the reaction temperature raises from 150 to 180 °C, a conversion jump can be seen, because of the desorption of product methanol from metallic centers is limited by the peripheral NaZSM-5 framework at lower temperatures, as revealed by results of methanol-TPD (Supplementary Fig. 5). At 180 °C, a selectivity to CH_3_OH larger than 98% is obtained at CO_2_ conversion of 10.2% and the selectivity to CO + CH_4_ is less than 2%. When the reaction temperature exceeds 180 °C, the increase in amount of CO and CH_4_ lowers the selectivity to CH_3_OH, driven by thermodynamics (Supplementary Fig. 1). With the pressure rising, as described in Fig. 4b, both the conversion of CO_2_ and the selectivity of CH_3_OH increase, due to the principle of Le Chatelier. Noteworthy, the effect of space velocity on the reaction is very interesting and it can be seen from the Fig. 4c that the CH_3_OH selectivity increases and the selectivity to CO + CH_4_ decreases with the space velocity increasing. It is a little strange but well elucidated by DFT calculations, which clearly show that the energy barrier of *CO desorption is much higher than hydrogenation of *CO to *CHO (see below).

The service life of catalyst is of great importance for industrialization. A fatal problem of the hydrogenation of CO_2_ reported to date is that the deactivation of the most of supported metal catalysts due to the sintering of active components at high temperatures^32^. For current Mo_3_S_4_@NaZSM-5 catalyst, the active component [Mo_3_S_4_]^n+^ is protected by the surrounding ZSM-5 zeolitic framework which is extremely stable at relatively low reaction temperature of 180 °C. It keeps catalytic performance, i.e., CO_2_ conversion >10% and at the same time the CH_3_OH selectivity >98%, without any reduction in continuous 1000 h on stream of CO_2_ hydrogenation (Fig. 4d, 180 °C, 4 MPa, 1200 mL g_cat_^-1^ h^-1^).

For comparison, the catalysts MoS_x_/NaZSM-5 and commercial MoS_2_ are also tested for the reaction and the specific experimental data are listed in Table 2. Interestingly, both of them exhibit very low catalytic activity for CO_2_ hydrogenation under the same reaction conditions. The Mo_3_S_4_@NaZSM-5 catalyst with active center of [Mo_3_S_4_]^n+^ and peripheral NaZSM-5 framework surrounding should be a suitable combination showing excellent catalytic performance for CO_2_ hydrogenation to CH_3_OH. The surrounding NaZSM-5 framework confines and stabilizes the cluster of [Mo_3_S_4_]^n+^ in zeolitic cages with a small space remained just right for adsorption and reaction of CO_2_ and H_2_ to methanol, as shown in Fig. 1d.

**Table 2:** Catalytic hydrogenation of CO_2_ over related catalysts

Catalyst	Conversion (%)^a^	Selectivity (%)			STY^b^ (mg g_cat_^−1^ h^−1^)
		CH_3_OH	CH_4_/C_2_	CO	
MoS_2_^c^	0.3	54	39/–	7	0.64
MoS_x_/NaZSM-5	0.4	63	28/–	9	1
Mo_3_S_4_@NaZSM-5	10.2	98	0.7/–	1.3	39.5
Mo_3_S_4_@HZSM-5^d^	6.4	62.5	5/24	8.5	15.7

Reaction conditions: 180 °C, 4 MPa, 1200 mL g_cat_^−1^ h^−1^ of GHSV.

^a^Single-pass conversion of CO_2_.

^b^Space-time yield of CH_3_OH.

^c^Commercial MoS_2_ powder, referring to 3 wt% Mo, mixed with NaZSM-5.

^d^Mo_3_S_4_@NaZSM-5 was prepared from Mo_3_S_4_@HZSM-5 by neutralization of proton with NaOH. Calculation methods are given in Methods section.

Over Mo_3_S_4_@HZSM-5 catalyst, the reaction of CO_2_/3H_2_ gives 6.4% conversion and 62.5% methanol selectivity (Table 1). Some CO and CH_4_ are detected, reflecting the change of electronic properties of [Mo_3_S_4_]^n+^ clusters modulated by the inner dielectric field of zeolite upon the replacement of Na^+^ by proton^33^. C_2_ hydrocarbons (ethylene and ethane) in selectivity of 24% in the product are obviously from the reaction of CH_3_OH at Brønsted acid of HZSM-5.

### Reaction mechanism of CO_2_ hydrogenation

**Fig. 5:**
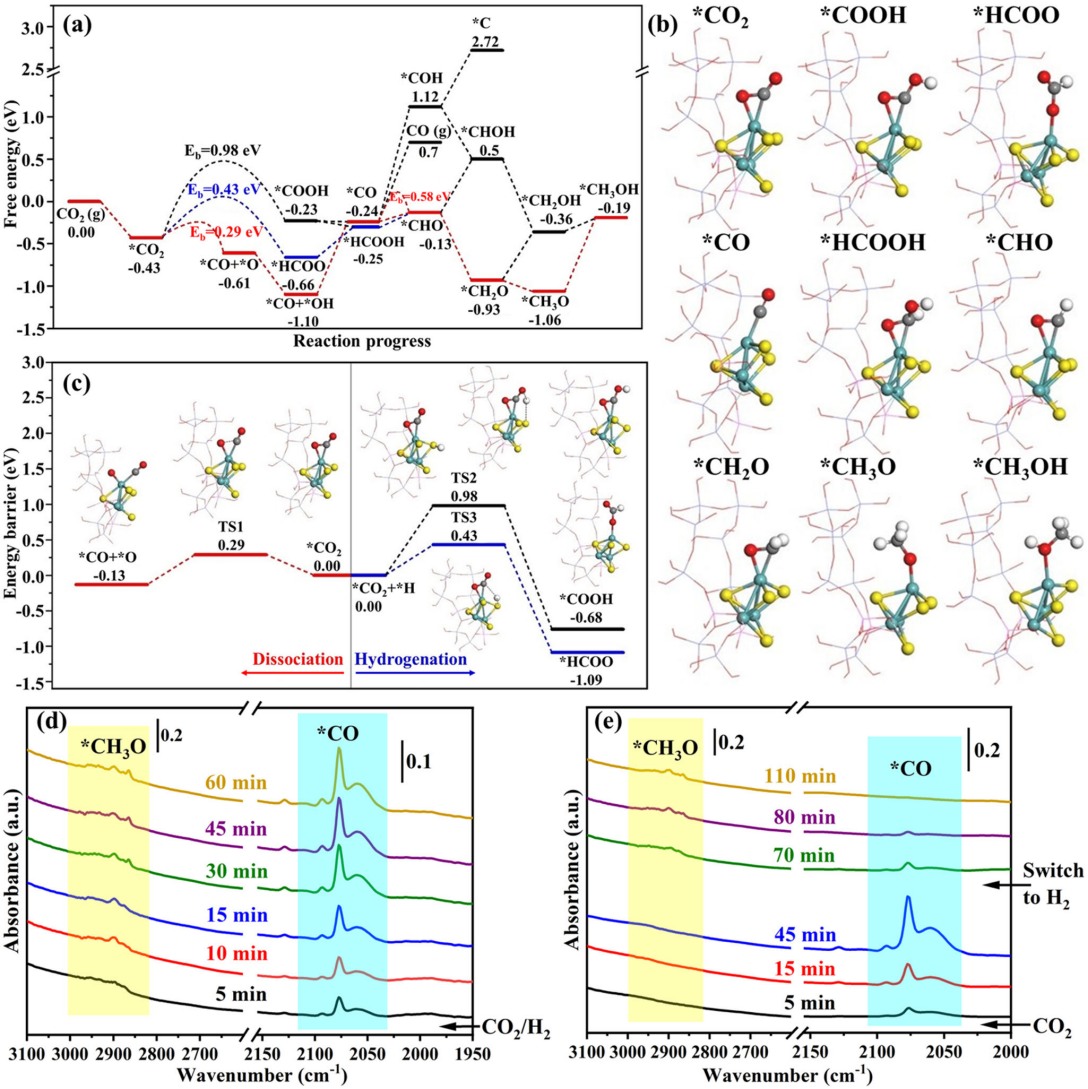
Study on the reaction mechanism of CO_2_ hydrogenation to CH_3_OH over Mo_3_S_4_@NaZSM-5. **a** Free energy diagram of CO_2_ hydrogenation to CH_3_OH over Mo_3_S_4_@NaZSM-5 catalyst and kinetic energy barrier (E_b_) of the key rate-determining step; **b** Possible intermediates for CO_2_ hydrogenation to CH_3_OH over Mo_3_S_4_@NaZSM-5; **c** Potential energy surface of *CO_2_ dissociation to *CO and hydrogenation to *COOH and *HCOOH over Mo_3_S_4_@NaZSM-5 catalyst; **d** Operando FT-IR spectra of Mo_3_S_4_@NaZSM-5 catalyst during the hydrogenation of CO_2_ under the reaction conditions (CO_2_/3H_2_, 4 MPa, 180 °C); **e** FT-IR spectrums of CO_2_ dissociation and after CO_2_ was switched to H_2_ at room temperature, 4 MPa by introducing feed gas into the chamber with a Mo_3_S_4_@NaZSM-5 catalyst disk mounted; The structural diagrams of intermediates and transition states (TS) for every step of reaction are shown in the inset. Color legend: Si (purple), Al (green), O (red), Mo (Cyan), S (yellow), and H (white).

Density functional theory (DFT) calculations were carried out to study the reaction mechanism of CO_2_ hydrogenation over Mo_3_S_4_@NaZSM-5 catalyst to understand the relationship between catalyst structure and catalytic activity at the atomic level. The thermodynamic route of CO_2_ hydrogenation to methanol and the kinetic energy barriers of key rate-determining steps are shown in Fig. 5a. The schematic diagram of the intermediate structure is shown in Fig. 5b. Firstly, CO_2_ is chemisorbed with C and O on Mo that does not bond with 10-MR in Mo_3_S_4_@NaZSM-5, forming *O-*C-O as shown in Fig. 5b. For this adsorption configuration, the hydrogenation of *CO_2_ to form *COOH or *HCOO competes with the direct dissociation of *CO_2_ to form *CO and *O formation. By comparing the activation energy barriers, we found that the activation energy barrier for the direct dissociation of *CO_2_ to *CO is 0.29 eV, while the activation energy barriers for *CO_2_ hydrogenation to *COOH and *HCOO are 0.98 and 0.43 eV respectively. The activation energy barrier for direct dissociation of *CO_2_ to *CO is much lower than that for the hydrogenation of *CO_2_ to *COOH and *HCOO, which is consistent with our experimental finding that the peaks of *CO was immediately captured in the infrared spectrum when the feeding gas with pure CO_2_ was introduced into a chamber in which a self-supported catalyst disk mounted for in-situ infrared measurement without H_2_ at room temperature (Fig. 5e). The potential energy surface and transition state structure of CO_2_ dissociation and hydrogenation are shown in Fig. 5c. The hydrogenation of *CO to *CHO requires remarkably lower activation energies than desorption from the surface of Mo_3_S_4_@NaZSM-5 catalyst. Importantly, the results also infer that the desorption of *CO, with activation barrier larger than 0.94 eV (0.70 + 0.24), is much difficult than its surface reaction of hydrogenation. The further hydrogenation of *CHO needs lower activation energies to generate CH_3_OH through *CH_2_O and *CH_3_O intermediates. Such reaction paths deduced by DFT calculations agree well with the results of operando FT-IR characterizations that the appearance of *CO and *CH_3_O signals as the feeding gas with CO_2_ and H_2_ were introduced into the chamber under typical reaction conditions as shown in Fig. 5d. Additionally, when CO_2_ was switched to H_2_ at room temperature, the peaks of *CO dissociated from CO_2_ gradually decreased until disappeared with the increase of *CH_3_O signals (Fig. 5e), which further proves that the reaction paths deduced from DFT calculations are completely reasonable. (The IR signals of *CO and *CH_3_O species are identified by Ref. ^22^).

### Catalytic conversion of syngas over Mo_3_S_4_@NaZSM-5 and Mo_3_S_4_@HZSM-5

We have also tested the catalytic conversion of syngas over catalysts of Mo_3_S_4_@HZSM-5 and Mo_3_S_4_@NaZSM-5 and the main results are listed in Table 3. Only CH_4_ and CH_3_OH are detected as organic products over Mo_3_S_4_@NaZSM-5. For Mo_3_S_4_@HZSM-5, however, C_2_ and C_3_ hydrocarbons are detected in organic products in 98% selectivity, besides 2% CH_4_, which obviously follows a cascade route, i.e., CO was firstly converted into CH_3_OH over [Mo_3_S_4_]^n+^ clusters and then CH_3_OH transformed into light olefins (most olefins are further hydrogenated to alkanes over [Mo_3_S_4_]^n+^ clusters) at the Brønsted acid of HZSM-5 zeolite.

**Table 3:** Catalytic syngas conversion over Mo_3_S_4_@NaZSM-5 and Mo_3_S_4_@HZSM-5

Feed gas	Catalyst	Conversion (%)^a^	Selectivity (%)		
			CH_3_OH	CH_4_ /C_2_-C_3_^b^	CO_2_
2CO/H_2_	Mo_3_S_4_@NaZSM-5	2.6	97.6	2.4/–	0.8
	Mo_3_S_4_@HZSM-5	3.2	–	2/98	45
CO/2H_2_	Mo_3_S_4_@NaZSM-5	5.4	81	19/–	7.6
	Mo_3_S_4_@HZSM-5	5.8	–	25/75	39

Reaction conditions: 260 °C, 4 MPa, 4,000 mL g_cat_^-1^ h^-1^ of GHSV.

^a^Single-pass conversion of CO.

^b^C_2_ -C_3_ hydrocarbons (including olefins and alkanes) selectivity in organic products. Calculation methods are given in Methods section.

**Fig. 6:**
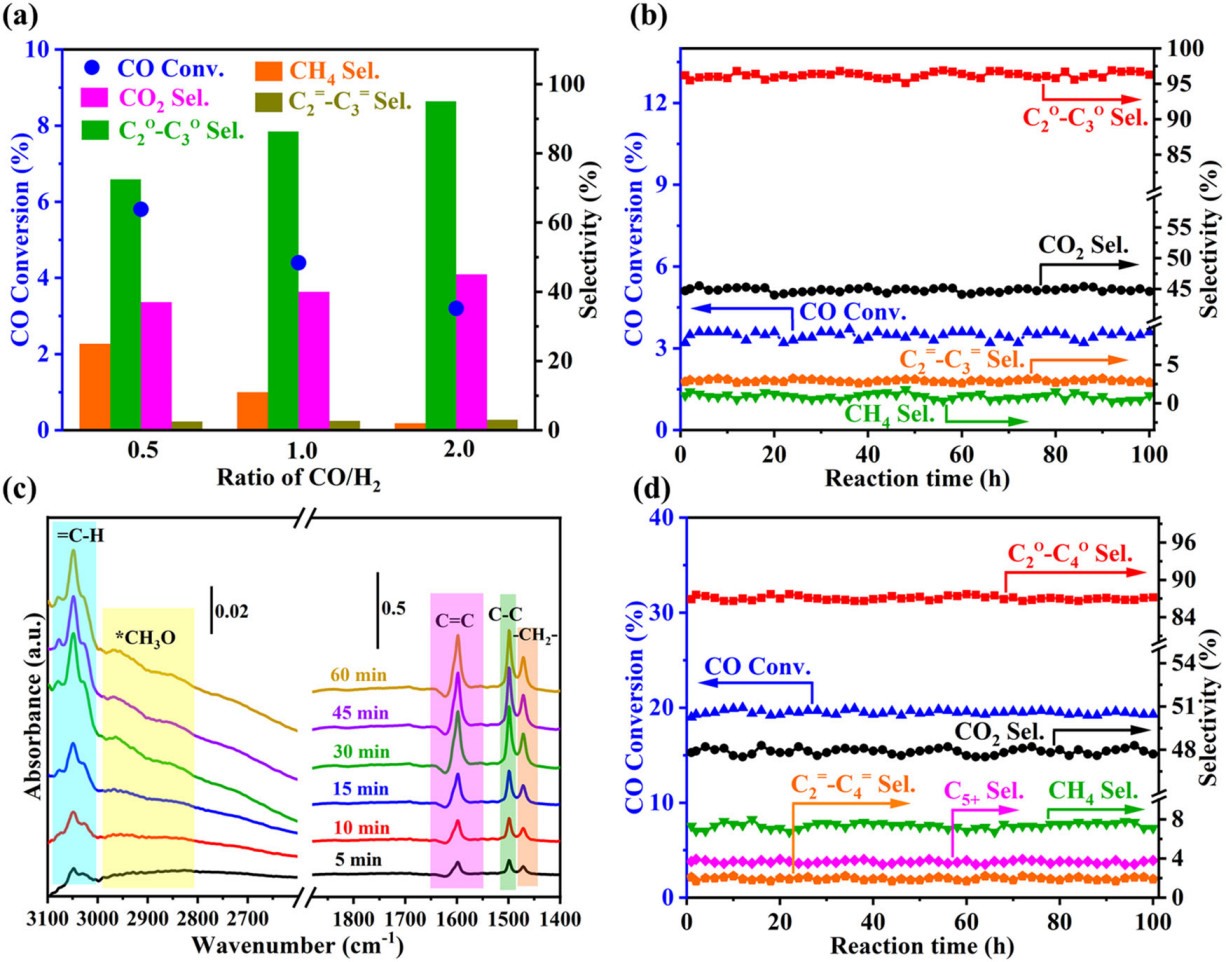
Syngas conversion over Mo_3_S_4_@HZSM-5. **a** Effect of CO/H_2_ ratio on CO conversion and product distribution (260 ^o^C, 4 MPa, 4,000 mL g_cat_^−1^ h^−1^); **b** Prolonged test of syngas conversion over Mo_3_S_4_@HZSM-5 (2CO/H_2_, 260 °C, 4 MPa, 4,000 mL g_cat_^−1^ h^−1^); **c** Operando FT-IR spectra of Mo_3_S_4_@HZSM-5 during the syngas conversion (2CO/H_2_, 260 °C, 4 MPa); **d** Prolonged test of syngas conversion over Mo_3_S_4_@HZSM-5 at elevated temperature (2CO/H_2_, 400 °C, 4 MPa, 4,000 mL g_cat_^−1^ h^−1^). Conv. and Sel. are abbreviations for Conversion and Selectivity, respectively. ^=^ refers to olefins, ^o^ refers to alkanes.

More details about the reaction of syngas conversion are shown in Fig. 6. Figure 6a shows that the selectivity of C_2_ + C_3_ hydrocarbons (olefins and alkanes) in organic products increased from 75% to 98% when the feed gas changed from CO/2H_2_ to 2CO/H_2_ and the CO conversion simultaneously decreased from 5.8% to 3.2%. It appears that the higher CO/H_2_ ratio benefits to improve the selectivity to light hydrocarbons. Furthermore, the catalytic performance keeps stable on stream more than 100 h with >98% C_2_ and C_3_ hydrocarbons in organic products and CO conversion > 3.2% with 2CO/H_2_ syngas, as shown in Fig. 6b. Figure 6c depicts the results of operando characterization of IR over Mo_3_S_4_@HZSM-5 during 2CO/H_2_ conversion (260 °C, 4 MPa). The species or intermediates of *CH_3_O, unsaturated C-H ( = CH), symmetric C = C stretching vibrations, C-C stretching vibrations^34^, and methylene bending band (-CH_2_-)^35^ can be observed during reaction. In addition, the absorption peak of CH_4_ at 3015 cm^-1^ did not appear, indicating that no CH_4_ formed during the syngas conversion at 260 °C, which is consistent with the results shown in Fig. 6b.

The catalytic stability of Mo_3_S_4_@HZSM-5 for syngas (2CO/H_2_) conversion is further tested at 400 °C for 100 h and the results are shown in Fig. 6d. C_4_ and small amount of C_5+_ hydrocarbons are detected in product. The selectivity of ~90% to C_2_-C_4_ hydrocarbons in the organic products is measured at CO conversion of ~20% and the results keep unchanged in 100 h on stream. The results deviate far from the ASF model that predicts the selectivity to C_2_-C_4_ hydrocarbons cannot exceed 58%. It is very important that the result of composition analysis on spent catalyst indicate the loss of S is negligible, even on stream of 100 h at 400 °C (Supplementary Table 1). The unique Mo_3_S_4_@ions-ZSM-5 catalyst, compliant with so-called mesocatalyst in structure we summarized previously^36^, would offer opportunities to discovery more interesting and important catalytic reactions and further works are under the way.

In summary, the catalyst Mo_3_S_4_@ions-ZSM-5 with [Mo_3_S_4_]^n+^ clusters embedded in the cages of ZSM-5 zeolite is obtained by two-step solid exchange or reaction method. The properties of the catalyst can be further modified by exchanging the remaining ions with other metallic ions. It is realized the catalyst is capable of dissociating CO_2_ at room temperature to CO adsorbed on the clusters. With the catalyst Mo_3_S_4_@NaZSM-5, the hydrogenation of CO_2_ in selectivity more than 98% to methanol and 10.2% conversion of CO_2_ at 180 °C is confirmed stable at least 1000 h on stream, without any decay. Furthermore, a related catalyst Mo_3_S_4_@HZSM-5 exhibits a peculiar result to catalyze the conversion of syngas with selectivity of C_2_ and C_3_ hydrocarbons more than 98% in organics at 260 °C, far beyond the limits of the ASF model. Even at 400 °C for 100 h on stream, the loss of Sulphur from Mo_3_S_4_@HZSM-5 catalyst is negligible. The significance of the catalyst, for conversion of CO_2_ or syngas, is considered originating from the special combination of the [Mo_3_S_4_]^n+^ center and the peripheral zeolitic framework. We believe such structural patterns are very effective to design high-performance catalysts.

## Methods

### Catalyst preparation

#### Mo_3_S_4_@HZSM-5

Firstly, 1.5 g HZSM-5 zeolite with the Si/Al molar ratio of 19 and 0.07 g MoO_3_ (Aladdin Reagent Co., Ltd., 99.9%) were physically mixed and ground in an agate mortar for half an hour. These mixtures were then placed in a tubular furnace and heated at a rate of 5 °C/min in a flowing air to 700 °C and maintained at 700 °C for half an hour. In the step, the MoO_3_ migrates into the channel of HZSM-5 zeolite and reacts with hydroxyl groups in the zeolite to form (Mo_2_O_5_)^2+^ dimer and to cling to ZSM-5 framework^18, 20^. Secondly, the Mo_2_O_5_@HZSM-5 was mixed with 1.9 g sulfur powder (Sigma-Aldrich Reagent Co., Ltd., 99.9%) and ground in an agate mortar again for half an hour. Then, the mixture was heated in flowing 10 vol% H_2_/N_2_ at 500 °C for 3 h. The obtained material was denoted as Mo_3_S_4_@HZSM-5 (Mo: 3.01 wt%, obtained from inductively coupled plasma-optical emission spectrometer, ICP-OES).

#### Mo_3_S_4_@NaZSM-5

The Mo_3_S_4_@HZSM-5 was stirred in 0.05 mol/L NaOH ethanol solution (containing 10% water) for one hour at room temperature. The precipitate was filtered out and washed 5 times with ethanol, then dried at 80 °C for 12 h in vacuum. The catalyst is denoted as Mo_3_S_4_@NaZSM-5.

#### MoS_x_/HZSM-5

By mixing 1.5 g HZSM-5 zeolite, 0.07 g MoO_3_, and 1.9 g sulfur powder together in an agate mortar and then heating at 500 °C for 3 h in flowing 10 vol% H_2_/N_2_. The Mo content in MoS_x_/HZSM-5 is 3.03 wt% (obtained by ICP-OES analysis).

#### MoS_x_/NaZSM-5

MoS_x_/NaZSM-5 was obtained by neutralization reaction of MoS_x_/HZSM-5 in 0.05 mol/L NaOH ethanol/water (9:1) solution.

### Hydrogenation of 2,3-dimethylnitrobenzene and nitrobenzene

Typically, 0.3 g catalyst Mo_3_S_4_@HZSM-5 or MoS_x_/HZSM-5, 1 mmol 2,3-dimethylnitrobenzene, 1 mmol nitrobenzene and 20 mL ethanol were added to a 50 mL Teflon-lined autoclave. The autoclave was then filled with 2 MPa of H_2_, after the replacement of air in the reactor for five times by hydrogen. Finally, the autoclave was heated at 100 °C for a period of time with a magnetic stirring rate of 400 r/min. The conversion of reactants and selectivity to products were determined by gas chromatography using p-xylene as internal standard.

#### Hydrogenation of CO_2_ in fixed bed reactor

Catalytic hydrogenation of CO_2_ was performed in a tubular continuous-flow, fixed-bed reactor equipped with a gas chromatograph (GC-9860). In a typical procedure, the catalyst was pressed and crushed to particles between 20 and 40 mesh. 0.5 g catalyst was then placed in a U-shaped reaction tube (316 L stainless steel) with an inner diameter of ~4 mm. A mixed gas (23% CO_2_, 69% H_2_ and balance Ar) was used to measure the catalytic property for hydrogenation of CO_2_. Products were analyzed using an on-line gas chromatography equipped with a FID detector and two thermal conductivity detectors (TCD). The Ar in feed gases was used as internal standard.

The conversion and selectivity were calculated as follows:

5 $${{{{{\rm{CO}}}}}}_{2}\,{{{{{\rm{Conversion}}}}}}=\frac{{{{\mbox{CO}}}}_{2{{\mbox{inlet}}}}-{{{\mbox{CO}}}}_{2{{\mbox{outlet}}}}}{{{{\mbox{CO}}}}_{2{{\mbox{inlet}}}}}\times 100\%$$

6 $${{{{{\rm{CH}}}}}}_{3}{{{{{\rm{OH\; Selectivity}}}}}}=\frac{{{{{\mbox{CH}}}}_{3}{{\mbox{OH}}}}_{{{\mbox{outlet}}}}} {{{{\mbox{CO}}}}_{{{\mbox{outlet}}}}+{{{\mbox{CH}}}_{4}}_{{{\mbox{outlet}}}}+{{{\mbox{CH}}}_{3}{{\mbox{OH}}}}_{{{\mbox{outlet}}}}}\times 100\%$$

7 $${{{{{\rm{CO\; Selectivity}}}}}}=\frac{{{{\mbox{CO}}}}_{{{\mbox{outlet}}}}}{{{{\mbox{CO}}}}_{{{\mbox{outlet}}}}+{{{\mbox{CH}}}_{4}}_{{{\mbox{outlet}}}}+{{{\mbox{CH}}}_{3}{{\mbox{OH}}}}_{{{\mbox{outlet}}}}}\times 100\%$$

8 $${{{{{\rm{CH}}}}}}_{4}\,{{{{{\rm{Selectivity}}}}}}=\frac{{{{\mbox{CH}}}_{4}}_{{{\mbox{outlet}}}}}{{{{\mbox{CO}}}}_{{{\mbox{outlet}}}}{{{{{\boldsymbol{+}}}}}}{{{\mbox{CH}}}_{4}}_{{{\mbox{outlet}}}}{{{{{\boldsymbol{+}}}}}}{{{\mbox{CH}}}_{3}{{\mbox{OH}}}}_{{{\mbox{outlet}}}}}\times 100\%$$

Where CO_2 inlet_ and CO_2 outlet_ respectively represent the amount of CO_2_ entering and flowing out of the reactor, referenced to inner standard argon. The CH_3_OH _outlet_, CO _outlet_ and CH_4 outlet_ denote the amount of CH_3_OH, CO and CH_4_ in product, referenced to inner standard argon.

For syngas conversion:

9 $${{{{{\rm{CO\; Conversion}}}}}}=\frac{{{{\mbox{CO}}}}_{{{\mbox{inlet}}}}-{{{\mbox{CO}}}}_{{{\mbox{outlet}}}}}{{{{\mbox{CO}}}}_{{{\mbox{inlet}}}}}\times 100\%$$

10 $${{{{{\rm{CO}}}}}}_{2}\,{{{{{\rm{Selectivity}}}}}}=\frac{{{{\mbox{CO}}}}_{2{{\mbox{outlet}}}}}{{{{\mbox{CO}}}}_{{{\mbox{inlet}}}}-{{{\mbox{CO}}}}_{{{\mbox{outlet}}}}}\times 100\%$$

11 $${{{{{{\rm{C}}}}}}_{{{{{\rm{a}}}}}}} {{{{\mbox{H}}}}_{{{{{{\rm{b}}}}}}}}\; {{{{{\rm{Selectivity}}}}}}=\frac{{{{{{{\rm{aC}}}}}}}_{{{{{{\rm{a}}}}}}}{{{{\mbox{H}}}}_{{{{{{\rm{b}}}}}}}}_{{{\mbox{outlet}}}}}{{\sum }_{1}^{{{{{{\rm{a}}}}}}}{{{{{{\rm{aC}}}}}}}_{{{{{{\rm{a}}}}}}}{{{{\mbox{H}}}}_{{{{{{\rm{b}}}}}}}}_{{{\mbox{outlet}}}}}\times 100\%$$

Where C_a_H_b_ Selectivity denotes the individual hydrocarbon selectivity in organic products. The carbon balance before and after the reaction exceeds 95%.

### Characterization techniques

ICP-OES Avio500 instrument produced by PerkinElmer Co., Ltd was used to analyze the composition of catalysts. Micromeritics ASAP 2010 analyzer was used to measure the specific surface area of samples at liquid nitrogen temperature. X-ray diffraction (XRD) data were collected at BL14B1 beamline of Shanghai Synchrotron Radiation Facility from 0.8° to 25°. X-ray photoelectron spectroscopy (XPS) and Electron Spectroscopy for Chemical Analysis (ESCA) measurements were performed on a PHI5000 Versa Probe XPS system equipped with Al Kα X-ray as exciting source, the results were measured by transferring the samples to the test chamber under vacuum. The XAS spectra were collected at BL14W1 beamline of Shanghai Synchrotron Radiation Facility, for analysis of XANES and EXAFS. HAADF-STEM characterization was performed on a Thermo Fisher Themis Z transmission electron microscope (operated at 300 kV). The STEM was equipped with a probe corrector, monochromator, HAADF detector, segmented DF4 detector and SuperX EDS system. This instrument enables us to obtain a spatial resolution of 60 pm under STEM mode. The high-resolution iDPC-STEM (acquired by four-quadrant segmented detectors) images and corresponding STEM-HADDF images were acquired simultaneously with a convergence semi-angle of 25 mrad. The HAADF and iDPC-STEM images were acquired at collection angles of 21–127 and 5–20 mrad, respectively. The range of beam current was 0.5–1.5 pA and no beam damage on zeolite was observed during the STEM characterization. The elemental mappings were acquired by SuperX EDS system.

CH_3_OH temperature-programmed desorption (CH_3_OH-TPD) was carried out on a TP5076 apparatus (Tianjin Xianquan industry and Trade Development Co., Ltd) equipped with a TCD. Operando Fourier transform infrared spectroscopy (FT-IR) techniques consisting of a Bruker TENSOR 27 spectrometer and an in-situ IR cell with CaF_2_ windows which can be heated and pressurized. Typically, ~10 mg of Mo_3_S_4_@NaZSM-5/Mo_3_S_4_@HZSM-5 catalyst was pressed to disk and placed in the IR cell and pretreated in high purity helium of 30 mL/min at 180/260 °C for one hour. Then the helium was switched to CO_2_/3H_2_ or 2CO/H_2_ and the pressure was raised to 4 MPa. Finally, the infrared spectra were recorded with interval of 5-min to 110 min.

### Computational methods

All spin polarized DFT calculations were performed with the Vienna Ab initio simulation package (VASP)^37–39^. The exchange correlation function was handled using the generalized gradient approximation (GGA) formulated by the Perdew-Burke-Ernzerhof (PBE)^40, 41^. The plane-wave basis set energy cutoff was set to 400 eV, and the Brillouin zone integrations were done using a 1 × 1 × 1 k-point mesh. The electronic self-consistency criterion was set to 10^-4 ^eV and a force convergence tolerance of 0.02 eV/Å.

To investigate CO_2_ reduction mechanism, we have taken the climbing-image nudged elastic band method (CI-NEB), and the nature of transition state was verified by only one imaginary frequency existing in vibrational normal mode. The convergence criteria were 1 × 10^-4 ^eV energy differences for solving for the electronic wave function for transition states (TS), and a force convergence tolerance of 0.02 eV/Å.

The Gibbs free energy ΔG of CO_2_ to form CH_3_OH is defined as ΔG = ΔE + ΔE_ZPE_−TΔS. There, ΔE, ΔE_ZPE_, and ΔS are the energy change, the zero-point energy (ZPE) change and entropy change of the reaction, respectively. The zero-point energy and entropy of all intermediates and gaseous molecules are obtained by using the VASPKIT code^42^.

### Computational models

The ZSM-5 zeolite model was constructed using a periodic MFI unit cell with an experimental lattice constant of 19.88 Å × 20.11 Å × 13.37 Å. Some of Si atoms were substituted by Al atoms, resulting in a Si/Al ratio of 19. As shown in Fig. 1b, we consider three distinct ring sites of the MFI zeolite framework, namely the zigzag ten-membered ring (10-MR), 8-MR, and 6-MR sites, for hosting [Mo_3_S_4_]^n+^ in Fig. 1c. Due to the ring sizes, [Mo_3_S_4_]^n+^ is hosted only on the large 10-MR site. For 10-MR, we take a rigid frame structure and O is saturated with H, as shown in Fig. 1d. All -OH are fixed while the rest of the atoms are released.

As shown in Fig. 1d, the Mo-Mo bond length will be elongated and the Mo-S bond length remains basically unchanged when [Mo_3_S_4_]^n+^ is intercalated into 10-MR, [Mo_3_S_4_]^n+^ forms chemical bonds with two oxygen atoms in 10-MR, and the bond lengths are 2.33 and 2.16 Å respectively. And these two oxygen atoms are the meta positions of 10-MR Oxygen, respectively, forms chemical bonds with Al.

## Supplementary information

Supplementary Information

Peer Review File

## Data Availability

Source data are provided with this paper.

## Source data

Source Data
