# Supplementary material for: COx hydrogenation to methanol and other hydrocarbons under mild conditions with Mo3S4@ZSM-5
Source: Nat Commun. 2023 Jan 31;14:513. doi: 10.1038/s41467-023-36259-9 (PMC9889347; doi:10.1038/s41467-023-36259-9)
Supplement: Supplementary file 1 — Supplementary Information [file 41467_2023_36259_MOESM1_ESM.pdf]

Supplementary Information for

CO_x Hydrogenation to Methanol and Other Hydrocarbons under Mild Conditions with Mo_3S_4@ZSM-5

Gui Liu[1], Pengfei Liu[2], Deming Meng[1], Taotao Zhao[1], Xiaofeng Qian[1], Qiang He[1], Xuefeng Guo[1], Jizhen Qi[3], Luming Peng[1], Nianhua Xue[1], Yan Zhu[1], Jingyuan Ma[4]*,Qiang Wang[2]*, Xi Liu[5]*, Liwei Chen[3,5] and Weiping Ding[1]*

[1] Key Lab of Mesoscopic Chemistry, School of Chemistry and Chemical Engineering, Nanjing University, Nanjing 210023, China

[2] Department of Applied Chemistry, School of Chemistry and Molecular Engineering, Nanjing Tech University, Nanjing, 211880, China

[3] i-Lab, CAS Centre for Excellence in Nanoscience, Suzhou Institute of Nano-Tech and Nano-Bionics, Chinese Academy of Sciences, Suzhou, 215123, P. R. China.

[4] Shanghai Synchrotron Radiation Facility, 239 Zhangheng Road, Pudong New District, Shanghai 201204, China

[5] School of Chemistry and Chemical, In-situ Centre for Physical Sciences, Frontiers Science Centre for Transformative Molecules, Shanghai Jiao Tong University, Shanghai, 200240, P. R. China

*Correspondence author: dingwp@nju.edu.cn; majingyuan@zjlab.org.cn; wangqiang@njtech.edu.cn; liuxi@sjtu.edu.cn

Supplementary Information includes:

Supplementary Figures 1-5

Supplementary Tables 1-3

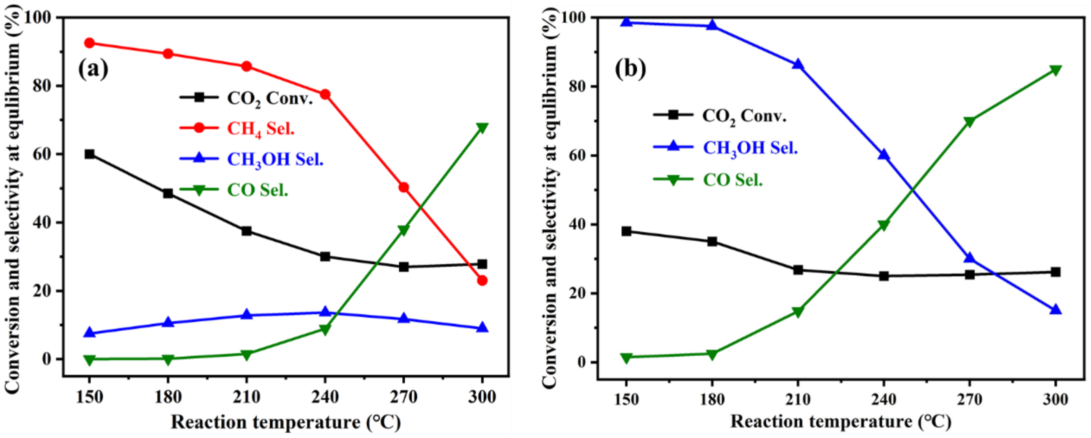

$$CO_2 + 3H_2 \rightarrow CH_3OH\ (g) + H_2O\ (g) \qquad \triangle H = -49.5\ kJ/mol \qquad (1)$$

$$CO_2 + H_2 \rightarrow CO + H_2O\ (g) \qquad \triangle H = 41.1\ kJ/mol \qquad (2)$$

$$CO_2 + 4H_2 \rightarrow CH_4 + 2H_2O\ (g) \qquad \triangle H = -164.9\ kJ/mol \qquad (3)$$

Supplementary Figure 1. The equilibrium values for hydrogenation of CO_2 to (a) CH_3OH, CO and CH_4 and (b) CH_3OH, CO in the reaction temperature range from 150 °C to 300 °C (Selectivity to water is not included).

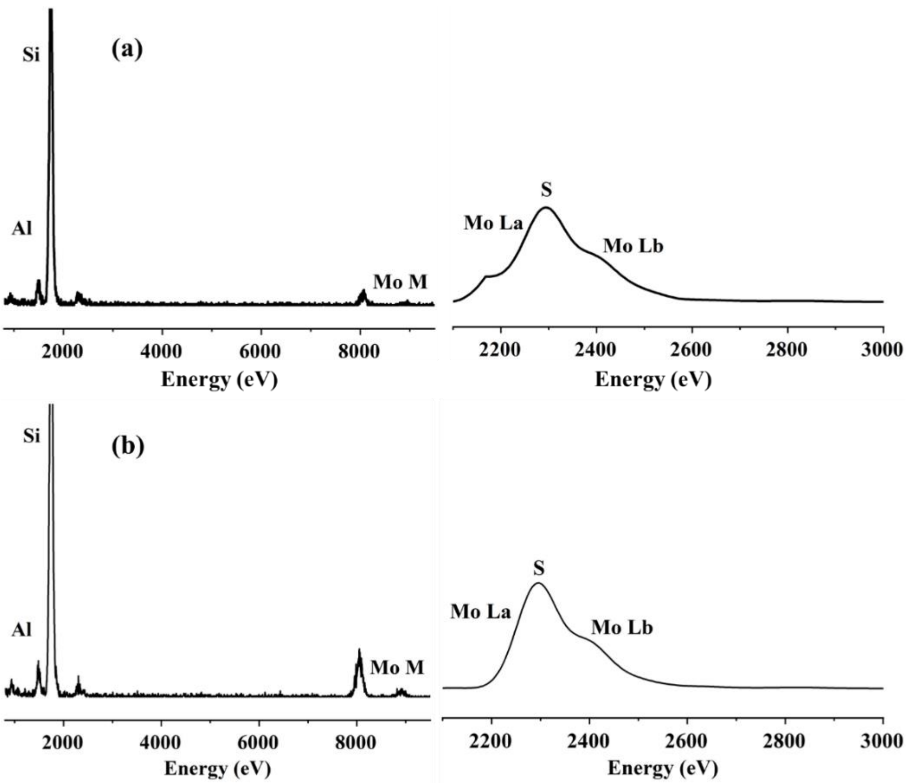

Supplementary Figure 2. The EDX profile of (a) fresh Mo_3S_4@NaZSM-5 and (b) spent sample obtained from the selected white square in Figure 2 (a-d).

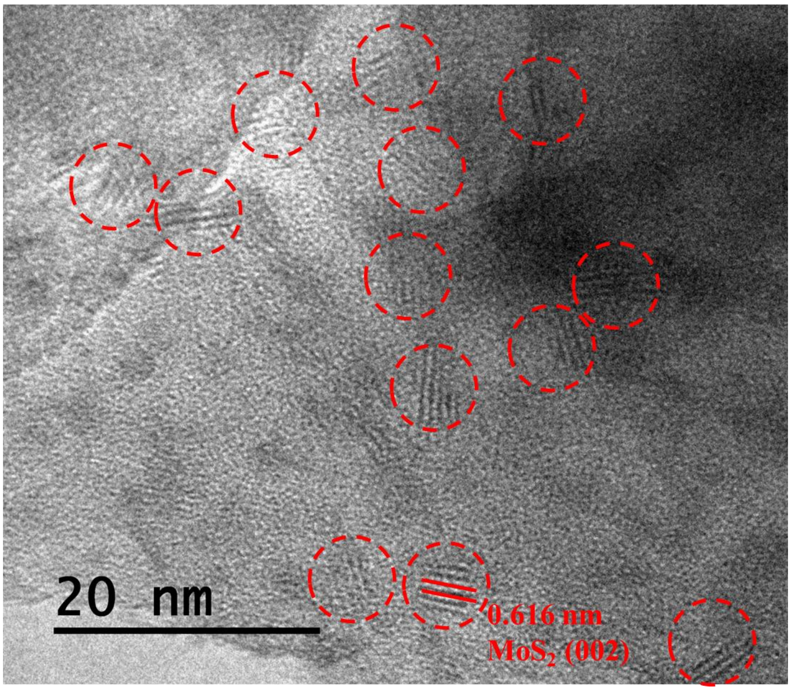

Supplementary Figure 3. The HRTEM image of MoS_x/NaZSM-5 catalyst.

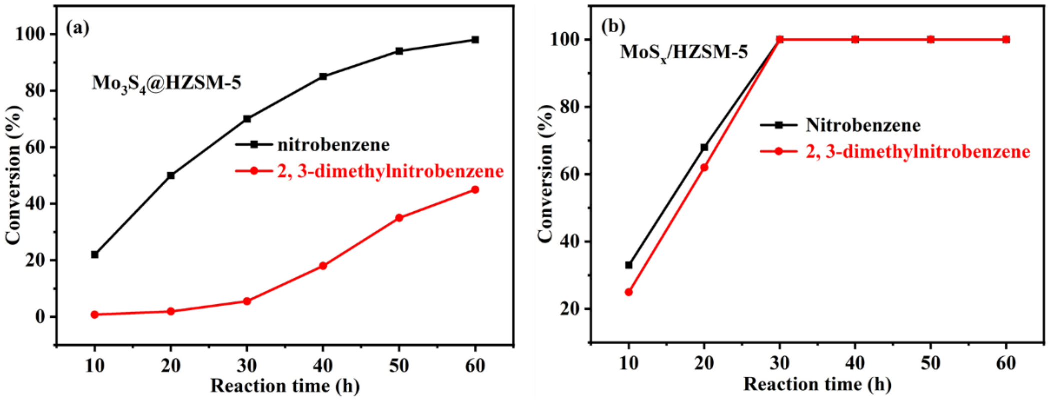

Supplementary Figure 4. Catalytic hydrogenation of the mixture of nitrobenzene and 2,3-dimethylnitrobenzene over (a) Mo_3S_4@HZSM-5 catalyst and (b) MoS_x/HZSM-5 catalyst (Reaction conditions: 0.3 g catalyst, 1 mmol nitrobenzene, 1 mmol 2,3-dimethylnitrobenzene, 20 mL ethanol, 2 MPa H_2. Data determined with a gas chromatography using p-xylene as internal standard.).

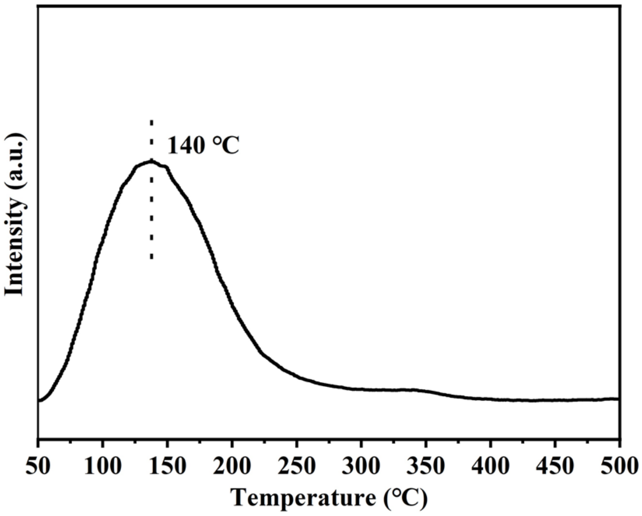

Supplementary Figure 5. CH_3OH temperature-programmed desorption over Mo_3S_4@NaZSM-5 catalyst.

Supplementary Table 1. S/Mo ratios of commercial MoS_2 mixed with ZSM-5, MoS_x/ZSM-5, Mo_3S_4@ZSM-5, and Spent Mo_3S_4@ZSM-5 analyzed by ESCA (Electron Spectroscopy for Chemical Analysis, the areas of binding energy peak are calibrated with Wager index for quantitative calculations).

Samples	Mole ratio of S/Mo
Commercial MoS_2+ZSM-5	1.90
MoS_x/ZSM-5	1.89
Mo_3S_4@ZSM-5	1.26
Spent Mo_3S_4@NaZSM-5 (at 180 °C)	1.22
Spent Mo_3S_4@HZSM-5 (at 400 °C)	1.21

Supplementary Table 2. Mo content (wt %) and BET specific surface area of various samples.

Samples	Mo (wt %)	BET specific surface area (m^2/g)
NaZSM-5	--	348.3
MoS_x/NaZSM-5	3.03	340.5
Mo_3S_4@NaZSM-5	3.01	270.7

Supplementary Table 3. Catalytic hydrogenation of 2,3-dimethylnitrobenzene and nitrobenzene over Mo_3S_4@HZSM-5 or MoS_x/HZSM-5 catalysts.

Catalyst	Product	Time (h)	Yield (%) [a]
Mo_3S_4@HZSM-5	aniline	60	98
	2,3-dimethyl-aniline		45
MoS_x/HZSM-5	aniline	30	>99
	2,3-dimethyl-aniline		>99

Reaction conditions: 0.3 g catalyst, 1 mmol nitrobenzene, 1 mmol 2,3-dimethylnitrobenzene, 20 mL ethanol, 2 MPa H_2. [a] Determined with gas chromatography using p-xylene as internal standard.
